# Development of novel multiplex microsatellite polymerase chain reactions to enable high-throughput population genetic studies of *Schistosoma haematobium*

**DOI:** 10.1186/s13071-015-1044-6

**Published:** 2015-08-20

**Authors:** B. L. Webster, M. Rabone, T. Pennance, A. M. Emery, F. Allan, A. Gouvras, S. Knopp, A. Garba, A. A. Hamidou, K. A. Mohammed, S. M. Ame, D. Rollinson, J. P. Webster

**Affiliations:** Wolfson Wellcome Biomedical Laboratories, Department of Life Sciences, Natural History Museum, Cromwell Road, London, SW7 5BD UK; Department of Infectious Disease Epidemiology, Imperial College Faculty of Medicine (St Mary’s Campus), Norfolk Place, London, W2 1PG UK; RVC Department of Pathology and Pathogen Biology, Centre for Emerging, Endemic and Exotic Diseases (CEEED), Royal Veterinary College, University of London, Hertsfordshire, AL97TA UK; Department of Epidemiology and Public Health, Swiss Tropical and Public Health Institute, Socinstrasse 57, 4002 Basel, Switzerland; University of Basel, Petersplatz 1, 4003 Basel, Switzerland; Réseau International Schistosomoses, Environnement, Aménagement et Lutte (RISEAL-Niger), 333, Avenue des Zarmakoye, B.P. 13724 Niamey, Niger; Public Health Laboratory - Ivo de Carneri (PHL-IdC), P.O.BOX 122, Wawi, Chake Chake, Pemba United Republic of Tanzania

**Keywords:** Cercariae, High-throughput, Microsatellites, Miracidia, Multiplex, Population genetics, *Schistosoma haematobium*

## Abstract

**Background:**

Human urogenital schistosomiasis caused by *Schistosoma haematobium* is widely distributed across Africa and is increasingly targeted for control and regional elimination. The development of new high-throughput, cost-effective molecular tools and approaches are needed to monitor and evaluate the impact of control programs on the parasite populations. Microsatellite loci are genetic markers that can be used to investigate how parasite populations change over time and in relation to external influences such as control interventions.

**Findings:**

Here, 18 existing *S. haematobium* microsatellite loci were optimised to enable simultaneous amplification across two novel multiplex microsatellite PCR’s, each containing nine loci. Methods were developed for the cost effective and rapid processing and microsatellite analysis of *S. haematobium* larval stages stored on Whatman-FTA cards and proved robust on miracidia and cercariae collected from Zanzibar and Niger.

**Conclusion:**

The development of these novel and robust multiplex microsatellite assays, in combination with an improved protocol to elute gDNA from Whatman-FTA fixed schistosome larval stages, enables the high-throughput population genetic analysis of *S. haematobium*. The molecular resources and protocols described here advance the way researchers can perform multi locus-based population genetic analyses of *S. haematobium* as part of the evaluation and monitoring of schistosomiasis control programmes.

**Electronic supplementary material:**

The online version of this article (doi:10.1186/s13071-015-1044-6) contains supplementary material, which is available to authorized users.

## Findings

### Introduction

Infection with the blood fluke *Schistosoma haematobium* causes human urogenital schistosomiasis throughout Africa, parts of the Middle East, Madagascar and the Indian Ocean Islands, with an estimated 110 million people infected [1]. Several efforts are underway to control morbidity and ultimately to eliminate *S. haematobium* infection predominantly through the large-scale administration of the drug praziquantel (PZQ) [1]. The development of new high-throughput, low cost, molecular tools and approaches are now imperative, not only to elucidate the epidemiology and evolution of schistosomiasis but also to monitor and evaluate the impact of progressing control programs [2]. Here we present an enhanced method enabling the high-throughput and cost effective preparation of gDNA from individual schistosome larval stages facilitating multi-loci genetic analysis together with two novel *S. haematobium* multiplex microsatellite PCRs. Microsatellite loci are highly variable DNA markers in widespread use within the schistosomiasis research community as they enable population-level analysis [3]. The principal drawback of microsatellite markers has been the cost and labour associated with the need to genotype multiple loci. Significant cost and timesaving can be achieved by developing multiplex PCR systems that amplify multiple microsatellite loci in single reactions. The methods outlined here facilitate the high-throughput microsatellite-based population genetic analyses of *S. haematobium*.

### Microsatellite multiplex design and optimisation

*S. haematobium* microsatellite loci were available from [4] and [3]. Loci that were di, tri or tetra-mer repeats, non-compound, robust and had multiplexing potential were selected for further optimisation. Eighteen loci were chosen in total (15 from [3] and three from [4], Table 1). Initially the functionality and specificity of all the primer pairs were confirmed by amplifying all the loci in singleplex 12.5 μl reactions using 10 ng of *S. haematobium* reference gDNA obtained from the Schistosomiasis Collection at the Natural History Museum (SCAN [5]) and the Type-it Microsatellite PCR Kit (Qiagen) according to the manufacturer’s protocol.

**Table 1:** Details of the 18 selected microsatellite loci and the characteristics of the two multiplex microsatellite PCR assays. Loci Sh1-15 are from Travis *et al.,* 2013 and Loci C102, C111 and C131 are from Gower *et al.,* 2011. For Niger *Ho* = 0.596, *He* = 0.609, for Pemba *Ho* = 0.599, *He* = 0.638. The overall *Ho* = 0.597, *He* = 0.623

Panel 1	Marker	Forward Primer 5'- 3'	Reverse Primer 5'- 3'	Dye	Size Range (bp)	Repeat	A	Niger		Zanzibar	
								*H*_o_	*H*_e_	*H*_o_	*H*_e_
Panel 1	C102	TGTCTCTGTGAATGACCGAAT	TTAGATGAATAATAATGTTGAAACCAC	VIC	184–199	ATT	6	0.42	0.37	0.02	0.02
	Sh1	GCATCCAATTTCGTACAC	CCACATTAGGCCAACAAG	VIC	245–284	AAT	13	0.76	0.72	0.84	0.80
	Sh14	GTCCTCCTTCCCTCTTTG	CACATTCGTCCTAGATATCG	NED	184–240	ACTC	15	0.94	0.85	0.86	0.88
	C131	CTTGTCATTTGGGCATTGTG	CATGGTGAGGTTCAAACGTG	NED	253–265	AAT	4	0.00	0.00	0.00	0.00
	Sh6	GGGATGTATGCAGACTTG	TTGTTTGGCTGCAGTAAC	NED	309–321	AAT	7	0.48	0.44	0.84	0.76
	Sh9	GCTGAGCTTGAGATTG	CTTCTGTCCCATCGATACC	6-FAM	197–227	AAT	11	0.46	0.76	0.46	0.86
	Sh3	GCTGAGCTTGAGATTG	CTTCTGTCCCATCGATACC	6-FAM	270–366	AAT	30	0.76	0.86	0.94	0.86
	C111	CCCTTGTCTTCAATGCGTTA	GAACGTCTAACTGGCGATCA	PET	201–225	ATT	9	0.74	0.67	0.76	0.68
	Sh7	TCCAAGCACCATTATCAAG	ACGGAAACTTGTTGAAATG	PET	293–311	AAT	7	0.46	0.62	0.42	0.48
Panel 2	Sh2	TTAGTGTGTTTGGCTTCAAC	CCTCGAATGAAATCCTGAC	NED	155–218	AAT	21	0.84	0.90	0.56	0.89
	Sh5	TGTGCACAAGAAAGATTAAATG	ACGACAATGTTGCAAGTTC	NED	263–314	AAT	16	0.78	0.81	0.36	0.48
	Sh13	GAGCAGCTATTTCGTATCG	ACCGTGGACAGTTCATCAG	6-FAM	163–211	AAT	17	0.78	0.72	0.68	0.64
	Sh4	CCCATCGCTGATATTAAAG	TCTAGTCGTCTTGGGATCC	6-FAM	268–313	AAT	13	0.84	0.78	0.72	0.79
	Sh10	CGCATGTCATACCTATCTCC	GCTTATCAGGCCTATCTCC	PET	183–207	AAT	9	0.18	0.34	0.74	0.70
	Sh12	CGTCTTAGTGAGCCAGATG	CTCGTGGACATCATCAG	PET	245–278	AAC	11	0.06	0.06	0.56	0.65
	Sh8	CTAAACTGGCAAGATTTC	CAACGTGCCTTTATTTC	PET	282–321	AAT	14	0.76	0.81	0.84	0.83
	Sh11	TTGGTTTAGAAATTACATCACC	CCAACAATATTAATGGACAGC	VIC	183–213	ATC	9	0.68	0.58	0.68	0.69
	Sh15	CTTTCAGTAGGATTTGTTG	CGACGTCAAGCACTGTAC	VIC	274–301	ATC	10	0.78	0.65	0.50	0.466

Panel = single mulitplex PCR. A = observed number of alleles. Dye = the fluorescent dye label of the forward primer (VIC = green, NED = yellow, 6-FAM = Blue, PET = red). *Ho* = observed heterozygosity, *He* = expected heterozygosity

The loci were successfully divided into two multiplex panels each incorporating nine loci that gave the maximum size difference between each locus and a maximum of four overlapping loci at any size range, together with minimal variance of the annealing temperature of all the primers (*T*_m_) (Table 1). Within each panel the forward primer for each locus was 5' labelled with a fluorescent reporter dye according to the 5-dye detection system. Overlapping fragments were assigned a different dye and the maximum distance was maintained between fragments labelled with the same dye to enable accurate identification. The multiplex microsatellite PCRs for each panel were carried out in 12.5 μl reactions using 10 ng of *S. haematobium* reference gDNA and the Type-it Microsatellite PCR Kit (Qiagen) according to the manufacturer’s protocol. Different *T*_m_ values were tested with the optimal *T*_m_ that gave uniform and specific amplification for all loci in each panel determined at 54 °C. Singleplex and multiplex amplicons were visualised on 3 % gel red agarose gels before 2 μl of 1: 50 dilutions were mixed with 0.35 μl of GS500Liz size standard (Applied Biosystems) before being denatured for 5 mins at 95 °C and injected at a 10 s injection speed into an Applied Biosystems 3130xl DNA Analyser. Allele peaks were visualised in Geneious version 6.1.4 (www.geneious.com [6]) using the microsatellite plugin. The multiplex PCRs proved robust giving identical peak scores in repeated reactions, in singleplex *versus* multiplex reactions, and significant stutter peaks, n-1 products and allelic drop-out were not observed.

### Multiplex PCR optimisation and application on field-collected S. haematobium miracidia and cercaria

A novel, high-throughput and cost effective non-wash Whatman-FTA alkaline DNA elution protocol has been developed which provides ~38 μl of eluted DNA from a single schistosome larval stage which has been fixed on a classic indicating Whatman-FTA card. This three-step protocol is very simple, quick and is suitable for multi-well processing. Individual larval DNA is alkaline eluted from a single 2.0 mm Whatman-FTA punch and subsequently neutralised, providing usable DNA for many downstream applications including microsatellite and fragment analysis, mitochondrial and nuclear DNA/gene amplification (http://www.gelifesciences.com). The solutions (1 and 2) needed for the DNA elution steps can be easily made with standard laboratory chemicals at an insignificant cost, especially compared to alternative DNA preparation methods.

Individual *S. haematobium* miracidia were collected directly from individual urine samples of infected children in Niger and Pemba Island (Zanzibar, United Republic of Tanzania [7]). *S. haematobium* cercariae were also obtained from naturally infected *Bulinus globosus* snails from Niger. All samples were collected and individually preserved on Whatman-FTA cards [8, 9].

DNA elutions were carried out in low profile 1.2 ml 96 square well storage microplates with 96 square well sealing cap mats which facilitates DNA elution. The 2.0 mm Whatman-FTA punch containing the DNA from a single larval stage was incubated at room temperature in 14 μl of Solution 1 (0.1 M NaOH, 0.3 mM EDTA, pH13.0) for 5 mins. Subsequently, 26 μl of Solution 2 (0.1 M Tris–HCl, pH7.0) was added, the mixture was pulse vortexed three times, incubated for a further ten minutes at room temperature and then pulse vortexed ten times. The eluted DNA was then transferred to a 96 well storage plate and either used immediately or stored at -20 °C for future use.

The two multiplex microsatellite PCRs were performed on each available sample in 12.5 μl reactions using 2 μl of the eluted DNA and the Type-it Microsatellite PCR Kit (Qiagen) according to the manufacturer’s protocol with the addition of 1.25 μl of the Type-it Microsatellite PCR Kit Q-Solution. Optimal cycling parameters were, an initial denaturing step of 95 °C for 5 mins followed by 32 cycles of 95 °C for 30 s, 54 °C for 90 s, 72 °C for 3 mins and followed by a final elongation step of 60 °C for 30 mins. Reactions were checked by 3 % agarose gel electrophoresis and then diluted 1 in 10 before being denatured and injected at an optimal speed of 12 s into the Applied Biosystems 3130xl DNA analyser for analysis.

Allele peaks were checked and edited using Geneious 6.1.4 (www.geneious.com [6]) before being placed into amplicon size “bins” and exported for analysis. Panel 1 and 2 allele data were compiled for each sample for analysis (Additional file 1: Table S1). Data were analysed from ten miracidia, from five children from Koutoukale Zeno (Lat. 13.680, Long. 1.738) in Niger, five children from Chambani school (Lat. 5.33457 Long. 39.77256) on Pemba Island, Zanzibar, United Republic of Tanzania and also from 16 cercariae from two infected *Bulinus* snails from Niger.

All loci amplified successfully with no significant stutter peaks or n-1 products. Whilst low peak height was often observed in the loci Sh7 (Panel 1) compared to the other loci and was lower in samples from Niger compared to Pemba, the data were still scorable. Genetic diversity indices were calculated using the program GenAlEx 6.5 [10] and the presence of null alleles and allele dropout was evaluated using Micro-Checker [11]. The numbers of alleles observed across the loci ranged from 2 to 33 with loci C131 being the least diverse. Higher genetic diversity was observed in the Pembamiracidial population compared to that from Niger (Table 1). Cercariae obtained from each individual snail had identical genotypes, showing they were clonal, derived from a single miracidium.

### Inter-species specificity

The cross-reactivity of the multiplex microsatellite PCRs was also assessed on *S. mansoni,* which causes intestinal schistosomiasis and is very common throughout Africa and can sometimes be found ectopically excreted in urine samples in endemic co-infection foci [12]. Singleplex and multiplex reactions were performed on 10 ng of reference gDNA from individual *S. mansoni* male worms obtained from the Schistosomiasis Collection at the Natural History Museum (SCAN [5]). Cross-reactivity was found to be low: seven loci failed to amplify, six gave low and/or non-specific amplification, two exhibited a size shift and only three among the total of 18 loci amplified well and were within the size range expected (Table 2).

**Table 2:** Cross reactivity of the two multiplex microsatellite PCR assays on *S. mansoni*

	Marker	Size Range (bp)
Panel 1	C102	allelic drop-out
	Sh1	245–284
	Sh14	low amplification
	C131	low amplification
	Sh6	309–321
	Sh9	low amplification
	Sh3	allelic drop-out
	C111	allelic drop-out
	Sh7	allelic drop-out
Panel 2	Sh2	allelic drop-out
	Sh5	low amplification
	Sh13	allelic drop-out
	Sh4	254
	Sh10	168
	Sh12	242–272
	Sh8	allelic drop-out
	Sh11	allelic drop-out
	Sh15	allelic drop-out

In conclusion, this study describes two novel robust and informative multiplex microsatellite assays enabling the simultaneous amplification of 18 individual loci; facilitating population genetic analysis of all *S. haematobium* life-cycle stages. Protocols are presented that facilitate high-throughput, and cost effective processing and robust genetic analysis of *S. haematobium* larval stages. Such tools can greatly assist large-scale population genetic analysis of human schistosome populations such as that now underway within the SCORE programme (http://score.uga.edu). The alkaline elution of larval schistosome DNA from Whatman-FTA stored samples is simple, quick, high-throughput and low cost, providing adequate amounts of gDNA preparations for multiple molecular analyses and repeats, significantly overcoming the limitations encountered from the standard Whatman-FTA preparations [2]. Additionally, the multiplexing of the microsatellite loci significantly reduces the resources associated with genotyping multiple microsatellite loci for analysis.

## Ethics statement

For the Niger sample collection, ethical approval was obtained from the St Mary’s Hospital Local Ethics Research Committee (part of the Imperial College London Research Ethics Committee (ICREC; (EC NO: 03.36. R&D No: 03/SB/033E)) in London, United Kingdom. For the Zanzibar sample collection, ethical approval was obtained from the Zanzibar Medical Research and Ethics Committee (ZAMREC, reference no. ZAMREC 0003/Sept/011) in Zanzibar, United Republic of Tanzania, the “Ethikkomission beider Basel” (EKBB, reference no. 236/11) in Basel, Switzerland, and the Institutional Review Board of the University of Georgia (project no. 2012-10138-0). Within both Niger and Zanzibar, all aspects of sample collections were carried out in the framework of the disease control activities implemented and approved by the local Ministry of Health (MoH) and adopted by regional and local administrative and health authorities. The study participants were informed about the study objectives and procedures. Written consent was obtained from parents prior to sample collection from children. Participation was voluntary and children could withdraw or be withdrawn from the study at any time without obligation. All children were offered PZQ (40 mg/kg single oral dose) treatment in the frame of the following school-based or community-wide treatment carried out by the MoH.

## Additional file

Additional file 1: Table S1. Allele sizes for all 18 loci for 50 miracidia from both Niger and Pemba (Zanzibar). (CSV 15 kb)

## Abbreviations

SCORE: Schistosomiasis Consortium for Operational Research and Evaluation
PCR: Polymerase chain reaction
gDNA: Genomic deoxyribonucleic acid
PZQ: Praziquantel
