# Erratum to: Development of novel multiplex microsatellite polymerase chain reactions to enable high-throughput population genetic studies of *Schistosoma haematobium*

**DOI:** 10.1186/s13071-015-1134-5

**Published:** 2015-10-09

**Authors:** B. L. Webster, M. Rabone, T. Pennance, A. M. Emery, F. Allan, A. Gouvras, S. Knopp, A. Garba, A. A. Hamidou, K. A. Mohammed, S. M. Ame, D. Rollinson, J. P. Webster

**Affiliations:** Wolfson Wellcome Biomedical Laboratories, Department of Life Sciences, Natural History Museum, Cromwell Road, London, SW7 5BD UK; Department of Infectious Disease Epidemiology, Imperial College Faculty of Medicine (St Mary’s Campus), Norfolk Place, London, W2 1PG UK; RVC Department of Pathology and Pathogen Biology, Centre for Emerging, Endemic and Exotic Diseases (CEEED), Royal Veterinary College, University of London, Hertsfordshire, AL97TA UK; Department of Epidemiology and Public Health, Swiss Tropical and Public Health Institute, Socinstrasse 57, Basel, 4002 Switzerland; University of Basel, Petersplatz 1, Basel, 4003 Switzerland; Réseau International Schistosomoses, Environnement, Aménagement et Lutte (RISEAL-Niger), 333, Avenue des Zarmakoye, Niamey, B.P. 13724, Niger; Public Health Laboratory - Ivo de Carneri (PHL-IdC), Wawi, Chake Chake, Pemba United Republic of Tanzania

Unfortunately, the original version of this article [1], contained a mistake. In Table 1, the primers for Sh6 and Sh9 were included incorrectly. Instead of GGGATGTATGCAGACTTG TTGTTTGGCTGCAGTAAC and GCTGAGCTTGAGATTG CTTCTGTCCCATCGATACC they should have been Sh6 Forward Primer GGTGGATTACGCAATAG, Sh6 Reverse Primer TTTAATCAACCGGGTGTC and Sh9 Forward Primer GGGATGTATGCAGACTTG, Sh9 Reverse Primer TTGTTTGGCTGCAGTAAC respectively.

**Table 1:** Details of the 18 selected microsatellite loci and the characteristics of the two multiplex microsatellite PCR assays. Loci Sh1-15 are from Travis *et al.,* 2013 and Loci C102, C111 and C131 are from Gower *et al.,* 2011. For Niger ***Ho*** = 0.596, ***He*** = 0.609, for Pemba ***Ho*** = 0.599, ***He*** = 0.638. The overall ***Ho*** = 0.597, ***He*** = 0.623

Panel 1	Marker	Forward Primer 5'- 3'	Reverse Primer 5'- 3'	Dye	Size Range (bp)	Repeat	A	Niger		Zanzibar	
								*H*_o_	*H*_e_	*H*_o_	*H*_e_
Panel 1	C102	TGTCTCTGTGAATGACCGAAT	TTAGATGAATAATAATGTTGAAACCAC	VIC	184-199	ATT	6	0.42	0.37	0.02	0.02
	Sh1	GCATCCAATTTCGTACAC	CCACATTAGGCCAACAAG	VIC	245-284	AAT	13	0.76	0.72	0.84	0.80
	Sh14	GTCCTCCTTCCCTCTTTG	CACATTCGTCCTAGATATCG	NED	184-240	ACTC	15	0.94	0.85	0.86	0.88
	C131	CTTGTCATTTGGGCATTGTG	CATGGTGAGGTTCAAACGTG	NED	253-265	AAT	4	0.00	0.00	0.00	0.00
	Sh6	GGTGGATTACGCAATAG	TTTAATCAACCGGGTGTC	NED	309-321	AAT	7	0.48	0.44	0.84	0.76
	Sh9	GGGATGTATGCAGACTTG	TTGTTTGGCTGCAGTAAC	6-FAM	197-227	AAT	11	0.46	0.76	0.46	0.86
	Sh3	GCTGAGCTTGAGATTG	CTTCTGTCCCATCGATACC	6-FAM	270-366	AAT	30	0.76	0.86	0.94	0.86
	C111	CCCTTGTCTTCAATGCGTTA	GAACGTCTAACTGGCGATCA	PET	201-225	ATT	9	0.74	0.67	0.76	0.68
	Sh7	TCCAAGCACCATTATCAAG	ACGGAAACTTGTTGAAATG	PET	293-311	AAT	7	0.46	0.62	0.42	0.48
Panel 2	Sh2	TTAGTGTGTTTGGCTTCAAC	CCTCGAATGAAATCCTGAC	NED	155-218	AAT	21	0.84	0.90	0.56	0.89
	Sh5	TGTGCACAAGAAAGATTAAATG	ACGACAATGTTGCAAGTTC	NED	263-314	AAT	16	0.78	0.81	0.36	0.48
	Sh13	GAGCAGCTATTTCGTATCG	ACCGTGGACAGTTCATCAG	6-FAM	163-211	AAT	17	0.78	0.72	0.68	0.64
	Sh4	CCCATCGCTGATATTAAAG	TCTAGTCGTCTTGGGATCC	6-FAM	268-313	AAT	13	0.84	0.78	0.72	0.79
	Sh10	CGCATGTCATACCTATCTCC	GCTTATCAGGCCTATCTCC	PET	183-207	AAT	9	0.18	0.34	0.74	0.70
	Sh12	CGTCTTAGTGAGCCAGATG	CTCGTGGACATCATCAG	PET	245-278	AAC	11	0.06	0.06	0.56	0.65
	Sh8	CTAAACTGGCAAGATTTC	CAACGTGCCTTTATTTC	PET	282-321	AAT	14	0.76	0.81	0.84	0.83
	Sh11	TTGGTTTAGAAATTACATCACC	CCAACAATATTAATGGACAGC	VIC	183-213	ATC	9	0.68	0.58	0.68	0.69
	Sh15	CTTTCAGTAGGATTTGTTG	CGACGTCAAGCACTGTAC	VIC	274-301	ATC	10	0.78	0.65	0.50	0.466

**Panel =** single mulitplex PCR. **A** = observed number of alleles. **Dye** = the fluorescent dye label of the forward primer (VIC = green, NED = yellow, 6-FAM = Blue, PET = red). ***Ho*** = observed heterozygosity, ***He*** = expected heterozygosity

A corrected version of Table 1 is included below.
